# Open Book on the Water Slide: A Case Series of APC2 Pelvic Ring Injuries from High-Energy Aquatic Accidents

**DOI:** 10.3390/jcm15051729

**Published:** 2026-02-25

**Authors:** Adeeb Algaith, Kapil Soni, Attila Mácsai, Lilla Sándor, Ákos Csonka, Endre Varga, Petra Hartmann

**Affiliations:** Department of Traumatology and Orthopaedics, University of Szeged, 6725 Szeged, Hungary; algaithadeeb@gmail.com (A.A.); kapil3112@hotmail.com (K.S.); macsai.attila@med.u-szeged.hu (A.M.); sandor.lilla.viktoria@med.u-szeged.hu (L.S.); csonka.akos@med.u-szeged.hu (Á.C.); varga.endre@med.u-szeged.hu (E.V.)

**Keywords:** pelvic fractures, open-book injury, anterior-posterior compression, pubic symphisis, sacroiliac joint, aquatic accidents, accidental injuries

## Abstract

**Background and Objectives**: Pelvic ring injuries with symphyseal disruption are classically associated with high-energy mechanisms such as motor vehicle collisions. Recently, waterslides have emerged as an underrecognized but distinct source of severe pelvic trauma. Waterslide-related pelvic trauma represents a distinct biomechanical entity characterized by a supine or semi-supine body position at splashdown, extreme forced hip abduction, asymmetric lower-limb positioning, and abrupt hydrodynamic deceleration. The high descent velocity, abrupt hydrodynamic deceleration, and forced hip abduction at water entry may combine to generate open-book-type pelvic injuries. Evidence guiding diagnosis and surgical management in this setting remains scarce. **Materials and Methods**: We retrospectively analyzed a consecutive series of adult patients sustaining waterslide-related anterior–posterior compression type II (APC2) pelvic ring injuries. Demographic data and the body mass index (BMI), fracture classification, surgical strategy, complications, and functional outcomes were reviewed. Only patients with complete imaging, operative records, and follow-up were included. **Results**: Four patients (38–72 years) met the inclusion criteria. All sustained rotationally unstable open-book pelvic injuries and were classified as APC2; three were AO/OTA 61B2.3 and one 61B3.3. All patients were overweight or obese (BMI 27.2–31.2). Pelvic binders provided an effective acute reduction in symphyseal diastasis; however, in one bilateral injury, CT imaging obtained with the binder in situ masked posterior ligamentous instability. Definitive surgical fixation was performed in all cases. Early mechanical failure occurred in two patients treated with short anterior symphyseal plate constructs. In the bilateral injury, isolated anterior fixation failed repeatedly until posterior sacroiliac stabilization was added. No deep infections or thromboembolic events occurred. Although two patients required short observational ICU stays, none were admitted for hemodynamic instability or pelvic bleeding. **Conclusions**: At 12-month follow-up, three patients achieved pain-free ambulation without assistive devices, while one patient required intermittent use of a single crutch; all patients regained independence in daily activities. Waterslide accidents represent a high-energy injury mechanism capable of producing severe APC2 pelvic disruptions, particularly in patients with an elevated BMI. Awareness of this mechanism and meticulous assessment of posterior stability are essential to avoid under-treatment and mechanical failure.

## 1. Introduction

Pelvic fractures represent approximately 3% of all skeletal injuries but account for a disproportionately high rate of morbidity and mortality due to their association with vascular injury and organ damage [1]. Among these, fractures involving the symphysis pubis, often resulting from high-energy blunt trauma, are critical due to their impact on pelvic stability and associated soft tissue injury [2]. The World Health Organization (WHO) reports that road traffic accidents are the leading cause of trauma-related injuries globally, with pelvic ring fractures commonly seen in high-impact collisions [3]. The incidence of pelvic fractures varies across different age groups. Among hospitalized trauma patients, the incidence ranges from 8% to 9.3%. In the general population, the age-adjusted incidence of pelvic fractures is about 198 per 100,000 persons per year, with higher rates observed in older adults due to factors like osteoporosis and low-energy falls [4,5]. These fractures are associated with significant morbidity and mortality. Unstable pelvic fractures have an in-hospital mortality rate of approximately 8.3%, and open pelvic fractures can have mortality rates as high as 45%. The severity of these injuries often necessitates comprehensive management strategies to address both the fracture and associated complications [6,7]. Anterior–posterior compression (APC) type I injuries, characterized by symphyseal diastasis less than 2.5 cm with intact posterior sacroiliac ligaments, are typically stable and managed nonoperatively. However, accurate classification using systems like Young–Burgess and AO/OTA is essential to distinguish them from more severe injuries and to guide appropriate treatment, thereby minimizing complications such as infection, hardware failure, and loss of fixation [8,9,10,11]. APC type II injuries, characterized by partial disruption of the posterior pelvic ring with symphyseal diastasis, require a precise diagnosis and often surgical intervention. Accurate classification using Young–Burgess and AO/OTA systems supports effective treatment planning. Early identification of the fracture type can guide the decision between conservative and surgical approaches, as well as minimizing complications such as infection, hardware failure, and loss of fixation [12,13].

Although APC injuries are classically associated with high-energy mechanisms, such as motor vehicle collisions, several recent reports have highlighted waterslides as a previously unrecognized cause of severe pelvic trauma. Unlike dashboard or crush mechanisms in motor vehicle collisions, the resisting force of water acts over milliseconds across a wide pelvic surface, generating a powerful external rotation moment during forced hip abduction at splashdown, which preferentially disrupts the anterior pelvic ring while partially sparing vertical stability. The cases described in the literature illustrate a consistent pattern: Fletcher et al. (2016) highlighted this risk by reporting three pelvic fractures, including one accompanied by a vaginal laceration, following descent from a 35-foot slide in Jamaica [14]. Similar mechanisms were observed in subsequent publications: Williamson et al. (2018) described pubic symphysis diastasis after a closed-flume ride without a raft [15], while Fletcher (2020) reported an obese woman who developed an open-book fracture complicated by pulmonary embolism after using a thrill slide [16]. Further evidence was provided by Shaath et al. (2021), who documented three APC-type injuries on the same Orlando attraction, all requiring operative fixation [17]. The most severe case to date was presented by Baldini-Garcia et al. (2022), involving a morbidly obese man who sustained a complex fracture with sacral dissociation after falling from a 30 m slide into shallow water [18]. Taken together, these reports underscore waterslides as a distinct yet underestimated injury mechanism, with a particular risk in overweight or obese individuals exposed to extreme velocities and forced hip abduction at pool entry. Based on our national registry data, APC-type pelvic ring injuries are predominantly associated with motor vehicle collisions or falls from a height; notably, during the study period at our institution, all waterslide-related pelvic injuries consistently resulted in APC type II disruptions. While previous reports describe a broader spectrum ranging from APC type I to complex APC type III-equivalent injuries, this uniform pattern directly motivated the present analysis and underscores the potential severity of the waterslide mechanism [15,18].

Given the descriptive nature of the available data, this study was designed as a case series with a literature-contextualized discussion. Accordingly, the aim of this study was to characterize the injury pattern, diagnostic pitfalls, surgical management strategies, and outcomes of waterslide-related pelvic ring injuries, and to highlight mechanism-specific considerations distinct from conventional high-energy trauma.

## 2. Materials and Methods

This retrospective case series from a Level I university trauma center critically examines the surgical management and complication profiles of patients who sustained high-energy trauma with water slide-related injuries. Patient selection was based on pelvic injuries resulting from the waterslide mechanism rather than a specific APC subtype, and only those with complete follow-up documentation were included, ensuring the reliability of outcome tracking.

Detailed parameters, including age, BMI, comorbidities (ASA classification), injury mechanism, associated injuries, time to surgery, intraoperative bleeding, and transfusion requirements, were recorded. Imaging studies were independently reviewed by both a trauma surgeon and a radiologist using preoperative CT scans (Patient 1: GE LightSpeed VCT scanner; Patient 2: Philips Brilliance iCT 256 system; Patients 3 and 4: GE Revolution Evo 64-slice CT scanner, GE HealthCare Technologies, Inc., Eindhoven, The Netherlands) and 3D reconstruction (Patients 1 and 2: Biotronics 3Dnet Medical Software, version 2.15; Patients 3 and 4: GE AW Server Client for Windows, version 3.2) to ensure the accurate classification of pelvic ring injuries. Functional recovery and complications were assessed using the patient record system (e-MedSolution/MedSol, T-Systems Hungary, Budapest, Hungary), and 12-month follow-up data were obtained from the national registry (Hungarian Experimental Polytrauma Registry (HEPR), https://mtrauma.hu/registry/politrauma/ accessed on 1 November 2024), allowing the standardized capture of early (infection, reoperation, transfusion) and late complications (implant failure, osteolysis, functional impairment), which were critically analyzed.

Written informed consent for participation and publication of anonymized clinical data and images was obtained from all patients in accordance with EQUATOR recommendations [19].

## 3. Results

### 3.1. Patient Demographics and Injury Characteristics

**Figure 1:**
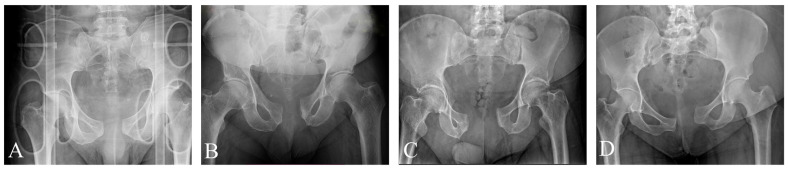
AP pelvic X-rays of patients upon admission. Patients 1 (**A**), 2 (**B**), and 4 (**D**) suffered unilateral injuries, coded as 61B2.3 according to the AO/OTA classification [10], while Patient 3 (**C**) suffered bilateral (61B3.3) injury, indicating bilateral involvement with posterior instability.

The study cohort consisted of four adult patients, aged 38 to 72 years (Table 1). All four patients sustained Anterior–posterior Compression Type II (APC2) pelvic ring disruptions characterized by significant symphyseal involvement. In one case, the skeletal injury was associated with a vaginal laceration. Radiographic assessment classified all injuries as “open book” fractures exhibiting rotational instability. No vertical shear displacement was observed initially. According to the AO/OTA classification, three cases were coded as 61B2.3 (unilateral injury), while one case was identified as 61B3.3 (bilateral injury with posterior instability). In this bilateral case, the deep posterior ligaments were assessed as partially intact, as no vertical migration was present (Figure 1 and Figure S1).

Body mass index (BMI) values for the cohort ranged from 27.2 to 31.2, indicating that all patients were overweight or obese. Injury Severity Scores (ISSs) ranged from 9 to 16, indicating moderate trauma. The patients presented with a low perioperative risk profile (ASA I–II) and no history of anticoagulant use (Table 1).

### 3.2. Treatment Strategies

**Figure 2:**
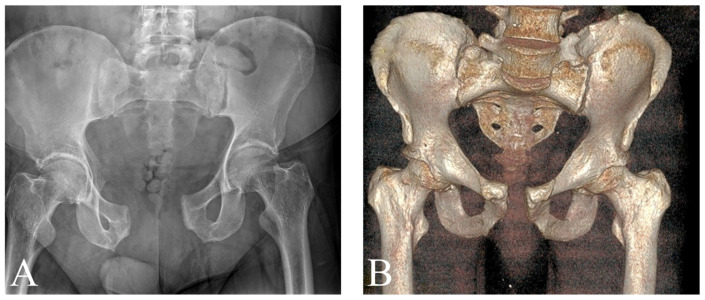
AP pelvic X-ray of Patient 3 upon arrival (**A**) and CT scan after placement of the binder (**B**). The X-ray shows AO/OTA 61B3.3 injury; the disrupted symphysis measures 60 mm. After application of the binder, the width of the symphysis reduces to 16 mm, and both sides of the SI joint seem anatomically aligned.

All cases received surgical treatment after application of a pelvic binder. The latter effectively reduced the pelvis [mean symphysis widening 44.48 mm, SD 13.81], which decreased by a mean of 20.98 [SD 5.31] (Figure 2).

**Figure 3:**
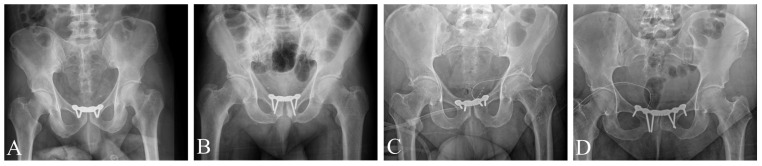
Postoperative AP pelvic X-rays. In all cases, low-profile stainless-steel reconstruction plates were used: 4-hole plates in Patients 1–3 (**A**–**C**), and a 6-hole plate in Patient 4 (**D**).

Definitive surgical fixation was performed in all cases. In two patients (Patients 1 and 4), the pelvic binder was exchanged for an anterior external fixator on the day of admission to maintain reduction. Conversion to open reduction and internal fixation (ORIF) of the pubic symphysis was performed between 1 and 6 days post-injury. Operative times ranged from 50 to 80 min. Fixation was achieved using 3.5 mm stainless-steel symphyseal reconstruction plates (Johnson Medtech). Three patients received a 4-hole plate construct, while one patient required a 6-hole plate (Figure 3).

Total hospital length of stay ranged from 11 to 19 days. Intraoperative blood loss varied notably, from 150 mL in the simplest case to 400 mL in the patient with bilateral injury (AO 61B3.3), who also required a blood transfusion (Table 2).

### 3.3. Complications

**Figure 4:**
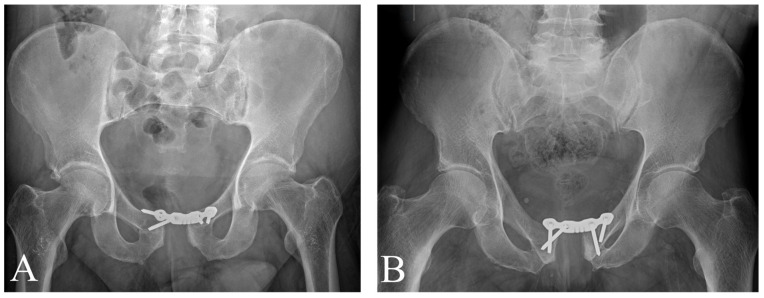
AP pelvic X-rays with early complications of Patient 2 (**A**) and Patient 3 (**B**).

Early mechanical failure of the fixation construct was observed in two of the four patients (Patients 2 and 3). Both had been treated with 4-hole reconstruction plates using angular-stable screws (Figure 4).

Patient 2 experienced loss of fixation at five weeks postoperatively. As a low-demand patient with minimal symptoms, he declined revision surgery.

**Figure 5:**
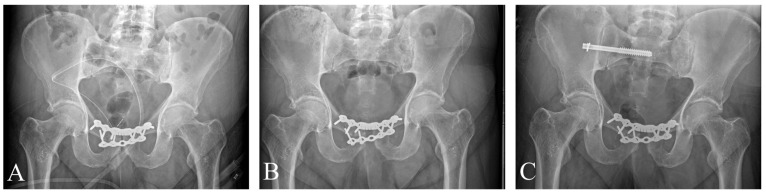
Revision surgeries of Patient 3. (**A**) First revision with double plating. The defect in the pubic rami was filled with bone graft harvested from the iliac crest, and the symphysis pubis was fixed with double plating. (**B**) After 60 days, signs of mechanical instability appeared, and the screws began to loosen. (**C**) An iliosacral screw insertion was used to stabilize the right SI joint. The posterior ring repair prevented any further mechanical complications.

Patient 3, who sustained the bilateral AO 61B3.3 injury, experienced a complex failure sequence. Initial preoperative planning relied on CT imaging performed with the pelvic binder in situ. This imaging masked the severity of the posterior ligamentous disruption. Consequently, the initial anterior-only fixation with a 4-hole plate failed on postoperative day 12 due to persistent posterior rotational instability. This necessitated two revision surgeries. The first revision involved orthogonal double plating of the symphysis and bone grafting. This construct also proved insufficient, showing signs of loosening. Definitive stability was ultimately achieved in a second revision via the insertion of a 10 mm titanium iliosacral screw to stabilize the right sacroiliac joint (Figure 5). Following posterior column stabilization, the patient became pain-free.

High-energy aquatic deceleration combined with an increased subcutaneous tissue mass may predispose patients to closed degloving injuries such as Morel–Lavallée lesions. Although none were clinically evident in our cohort, this entity should be actively considered in waterslide-related pelvic trauma, particularly in obese patients [20]. No deep surgical site infections or deep vein thromboses (DVT) were recorded. Two patients required short-term ICU admission for postoperative monitoring; however, no ICU stay was related to hemodynamic instability, pelvic bleeding, or trauma-related organ failure. Late complications were minor; two patients underwent elective hardware removal approximately one year post-injury.

### 3.4. Functional Outcomes

Functional recovery was assessed at 12 months post-injury using the Glasgow Outcome Scale Extended (GOS-E) [21]. Although GOS-E is not pelvis-specific and was originally developed for traumatic brain injury, it was used to provide a global functional assessment. This represents a limitation, and future studies should incorporate validated pelvic outcome scores such as the Majeed or Iowa Pelvic Score. Outcomes were favorable across the cohort. At the 12-month follow-up, three patients demonstrated pain-free ambulation without assistive devices, whereas one patient required intermittent use of a single crutch and was classified as having moderate disability (GOS-E score 6). No sexual dysfunction was reported. Full weight-bearing was typically authorized between 8 and 12 weeks postoperatively (Table 3).

## 4. Discussion

To our knowledge, this is the first case series to systematically describe pelvic ring injuries sustained during waterslide-related accidents and to characterize this mechanism as a distinct high-energy cause of pelvic trauma. The consistent occurrence of rotationally unstable open-book-type injuries highlights a previously underrecognized injury pattern with direct implications for diagnosis, fixation strategy, and failure prevention.

The outcomes observed in this cohort support the concept that waterslide-related pelvic injuries share key biomechanical characteristics with established high-energy injury mechanisms. While typically associated with motor vehicle collisions, the “waterslide” mechanism, characterized by high-velocity descent, rapid hydrodynamic deceleration, and forced hip abduction, represents a distinct and reproducible pathway to severe pelvic ring instability.

The Obesity Factor and Injury Severity: A notable characteristic of this cohort was the uniform elevation in body mass index (BMI > 27), with all patients classified as overweight or obese. This observation aligns with the recent literature by Coleman et al. (2020) [22], which demonstrates a significant association between the Young–Burgess injury classification and the need for urgent intervention in higher-BMI populations. In the context of waterslides, an increased body mass likely amplifies the kinetic energy transferred to the pelvis during the deceleration phase, potentially exacerbating the “open-book” distracting forces upon water entry.

Diagnostic Pitfalls: The Pelvic Binder. While pelvic binders proved highly effective for acute reduction, this series highlights a critical diagnostic caveat. In Patient 3, CT imaging acquired with the binder in place artificially reduced the posterior sacroiliac complex, masking the extent of ligamentous instability. This “false stability” led to the selection of an inadequate anterior-only fixation strategy, which ultimately failed. This reinforces the clinical recommendation that preoperative planning for high-energy APC injuries must critically evaluate posterior stability, potentially requiring an examination under anesthesia or stress views once the binder is released.

Fixation Strategies and Functional Outcomes: The failure of anterior-only plating in the patient with bilateral (61B3.3) injury underscores that restoring rotational stability in complex cases often requires addressing the posterior tension band. Despite these challenges, long-term functional outcomes were encouraging. The GOS-E scores at 12 months reflected a return to pre-injury independence for all patients. These findings are consistent with benchmarks for severe pelvic trauma established by Gabbe et al. (2015) [21], which suggest that favorable return-to-work and functional statuses are achievable with appropriate classification-based management. The absence of reported sexual dysfunction in this series is a positive deviation from historical rates, though larger cohorts are required for validation.

## 5. Conclusions

Waterslide accidents represent a high-energy mechanism capable of producing severe APC type II disruptions, particularly in patients with an elevated BMI. Based on this observation, biomechanical analyses and collaboration with water park safety authorities may help identify high-risk waterslide design features and patient-related risk factors, thereby informing targeted prevention strategies. Early recognition of posterior instability, which may be masked by the use of an acute pelvic binder, is crucial. Individualized surgical management and a high index of suspicion for posterior ligamentous injury are essential to prevent mechanical failure and ensure optimal long-term recovery. In this context, future multicenter studies are needed to establish injury-specific fixation algorithms and to validate posterior ring stabilization strategies in waterslide-related pelvic trauma.

## Figures and Tables

**Table 1:** Patient characteristics.

Case	1.	2.	3.	4.
Patient characteristics				
Age (year)	40	56	73	43
Sex (female/male)	male	male	male	female
BMI	27.17	29.01	31.01	31.22
Injury characteristics				
ISS	16	9	16	9
PMHx (ASA)	ASA1	ASA2	ASA2	ASA2
Classification				
Fracture classification (Y/B)	APC2	APC2	APC2	APC2
AO/OTA classification	61B2.3	61B2.3	61B3.3	61B2.3
PS widening without binder (mm)	40	35	60	38
PS widening with binder (mm)	22	18	16	18
PS widening with external fixator (mm)	9	N/A	N/A	21
SI displacement (mm)	0	6	10	8
Side of SI joint injury	N/A	N/A	Bilateral	N/A

ISS: Injury Severity Scores; PMHx: past medical history; ASA: American Society of Anesthesiologists physical status classification system; Y/B: Young–Burgess classification of pelvic fractures; PS: pubic symphysis; SI: iliosacral displacement.

**Table 2:** Operative and hospital course.

Case	1.	2.	3.	4.
Treatment	ExFix./ 4-hole plate	4-hole plate	4-hole plate	ExFix./ 6-hole plate
Time to external fixation (days)	0	N/A	N/A	0
Time to ORIF (days)	6	2	1	5
ORIF duration (min)	50	70	80	65
Intraoperative bleeding (mL)	150	250	400	300
Transfusion (units)	3	0	0	2
Revision surgery	None	None	Yes	None
Duration of revision surgery (min)	N/A	N/A	125	N/A

Operative and hospital course, ORIF.

**Table 3:** Clinical and surgical details and functional outcomes.

Case	1.	2.	3.	4.
Complications	None	Screws loosening 5 weeks postop	Screws loosening 12 days postop	None
Metal removal	Yes (1 year)	No	No	Yes (1 year)
ICU stay (days)	0	0	2	5
Hospital stay (days)	15	11	16	19
Sexual impairments	No	No	No	No
Walking aid	No	No	One crutch	No
GOSE	8	7	6	8

Clinical and surgical details and functional outcomes. ICU, intensive care unit. GOSE: The Glasgow Outcome Scale Extended (GOSE) is a global functional scale used to assess recovery and disability in patients. Values range from 8 (good recovery) to 7 (upper moderate disability) [21].

## Data Availability

All the data are available within the article.

## Supplementary Materials

The following supporting information can be downloaded at https://www.mdpi.com/article/10.3390/jcm15051729/s1, Figure S1: Preoperative CT imaging and 3D reconstruction. Representative preoperative imaging findings for all patients. (A) Axial preoperative CT scan. (B) Corresponding 3D reconstruction generated from the same CT dataset.

## Abbreviations

The following abbreviations are used in this manuscript:

APC2	Anterior–posterior compression type II
BMI	Body mass index
WHO	World Health Organization
APC	Anterior–posterior compression
ISS	Injury Severity Score
PMHx	Past medical history
ASA	American Society of Anesthesiologists Physical status classification system
Y/B	Young–Burgess classification of pelvic fractures
PS	Pubic symphysis
SI	Iliosacral displacement
DVT	Deep vein thromboses
ICU	Intensive care unit
GOS-E	Glasgow Outcome Scale Extended
