# Supplementary material for: Open Book on the Water Slide: A Case Series of APC2 Pelvic Ring Injuries from High-Energy Aquatic Accidents
Source: J Clin Med. 2026 Feb 25;15(5):1729. doi: 10.3390/jcm15051729 (PMC12986503; doi:10.3390/jcm15051729)
Supplement: Supplementary file 1 [file jcm-15-01729-s001.zip › jcm-4124989-supplementary.pdf]

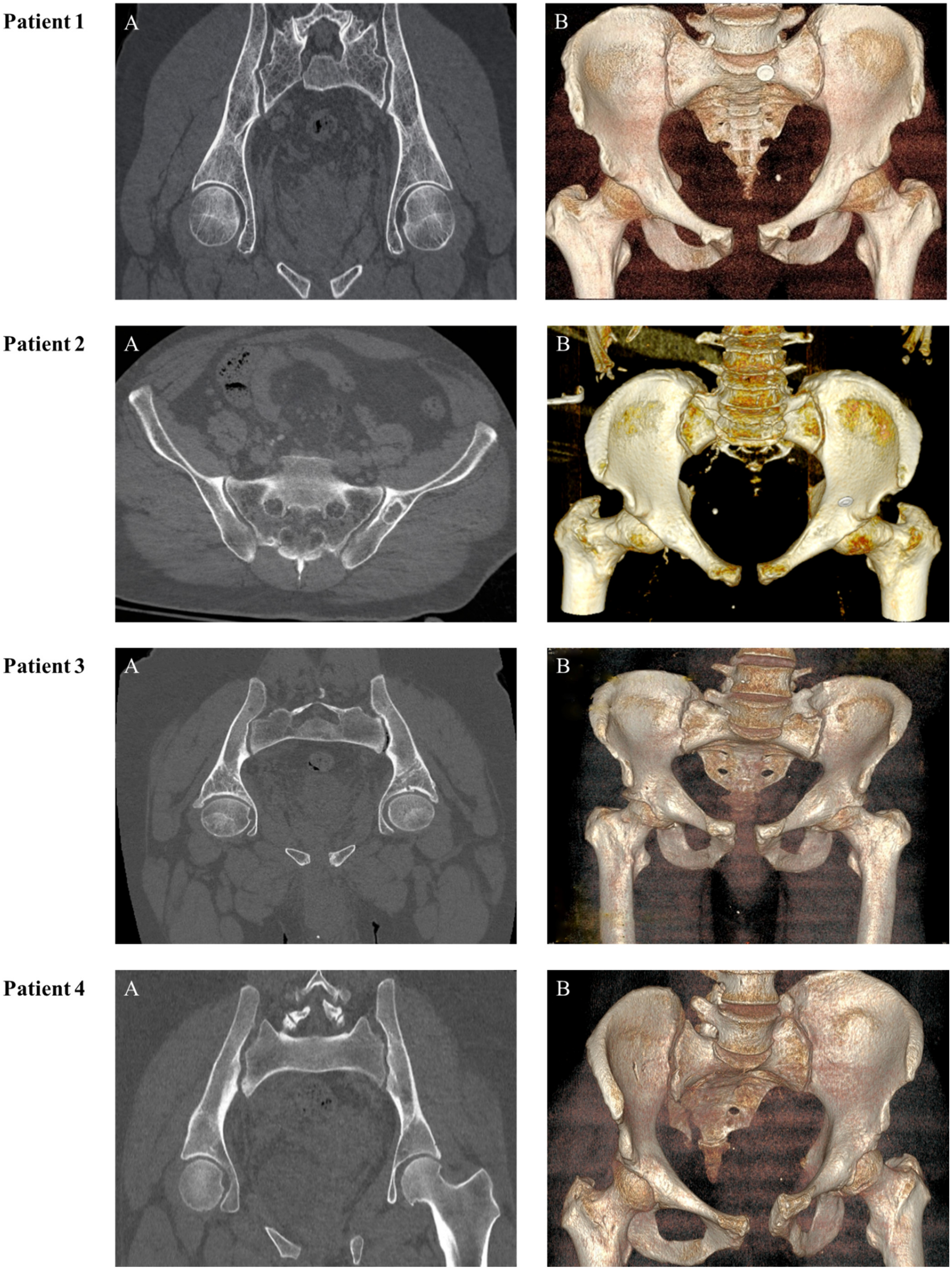

Supplementary Figure S1. Preoperative CT imaging and 3D reconstruction.

Representative preoperative imaging findings for all patients. (A) Axial preoperative CT scan. (B) Corresponding 3D reconstruction generated from the same CT dataset
